# Effect of different orthotic materials on plantar pressures: a systematic review

**DOI:** 10.1186/s13047-020-00401-3

**Published:** 2020-06-11

**Authors:** James M. Gerrard, Daniel R. Bonanno, Glen A. Whittaker, Karl B. Landorf

**Affiliations:** 1 Discipline of Podiatry, School of Allied Health, Human Services and Sport, La Trobe University, Melbourne, VIC 3086 Australia; 2 La Trobe Sport and Exercise Medicine Research Centre, School of Allied Health, Human Services and Sport, La Trobe University, Melbourne, 3086 Australia

**Keywords:** Orthotic device, Orthosis, Orthoses, Foot, Biomechanics, Kinetics, Plantar pressure

## Abstract

**Background:**

The effect of different orthotic materials on plantar pressures has not been systematically investigated. This study aimed to review and summarise the findings from studies that have evaluated the effect of orthotic materials on plantar pressures.

**Methods:**

We conducted a systematic review of experimental studies that evaluated the effect of foot orthotic materials or shoe insole materials on plantar pressures using in-shoe testing during walking. The following databases were searched: MEDLINE, CINAHL, Embase and SPORTDiscus. Included studies were assessed for methodological quality using a modified Quality Index. Peak pressure, pressure-time integral, maximum force, force-time integral, contact area, and contact time were variables of interest. Data were synthesised descriptively as studies were not sufficiently homogeneous to conduct meta-analysis. Standardised mean differences (Cohen’s *d*) were calculated to provide the size of the effect between materials found in each study.

**Results:**

Five studies were identified as meeting the eligibility criteria. All five studies were laboratory-based and used a repeated measures design. The quality of the studies varied with scores ranging between 20 and 23 on the modified Quality Index (maximum index score 28). The included studies investigated the effects of polyurethane (including PORON®), polyethylene (including Plastazote®), ethyl vinyl acetate (EVA) and carbon graphite on plantar pressures. Polyurethane (including PORON®), polyethylene (including Plastazote®) and EVA were all found to reduce peak pressure.

**Conclusion:**

Based on the limited evidence supplied from the five studies included in this review, some orthotic materials can reduce plantar pressures during walking. Polyurethane (including PORON®), polyethylene (including Plastazote®) and EVA reduce peak pressure beneath varying regions of the foot. Future well-designed studies will strengthen this evidence.

## Introduction

Foot orthoses and shock-absorbing insoles are commonly used to treat and prevent many foot and foot-related conditions [1, 2]. Accordingly, it is imperative that clinicians prescribing foot orthoses have robust evidence on the effects of orthotic materials to inform clinical decision-making. This is particularly pressing given the forecast of rapid growth within the foot orthotic industry [3]. Evidence should be based on findings from two sources: (i) well-designed randomised trials that evaluate *effectiveness* based on patient-based outcomes, and (ii) rigorous laboratory-based studies that assess the *effects* of materials on key biomechanical variables that are linked to effectiveness (i.e. surrogate outcomes) [4].

Regarding laboratory-based outcomes, one of the key effects of orthotic materials on the foot is the influence on plantar pressures [5–7]. Materials can reduce plantar pressure by reducing force or increasing contact area, or both. Therefore, materials can influence plantar pressures via their effects on different variables (e.g. force and contact area), which is likely to be dependent on their hardness, density, thickness and ability to contour to the foot. Early research that assessed the effects of orthotic materials was limited to bench-top testing using engineering instrumentation for mechanical analysis (such as a durometer measuring apparatus to assess hardness [8]) and force plate investigations [9], or a combination of the two. However, ‘in-shoe’ testing using pressure measuring insoles has now become commonplace to examine the effect of orthotic materials on pressure-related variables during gait.

Ideally, standardised results of such ‘in-shoe’ plantar pressure testing (i.e. from studies that used the same experimental conditions, so they can be directly compared and data pooled in meta-analyses) should be widely available for commonly used orthotic materials. Such data could be used by clinicians when prescribing foot orthoses for patients. For example, it would be highly worthwhile having valid data that is readily available to clinicians (e.g. freely available online) that presents the plantar pressure reductions that commonly used orthotic materials offer, including different thicknesses of those materials. This would enable clinicians the ability to consult such a source to inform their decision-making regarding orthotic materials for patients. Once robust data is available, guidelines could be developed for the materials that foot orthoses are constructed from, so consistent advice is provided to professions that use foot orthoses.

With the above in mind, it is timely to review and summarise the effects of different foot orthotic materials on plantar pressures. The aim of this study was to conduct a systematic review of the current literature by critically evaluating and summarising relevant studies that have assessed the effects of different foot orthotic materials on in-shoe plantar pressures during walking.

## Methods

### Search strategy

This systematic review is reported in accordance with the Preferred Reporting Items for Systematic Reviews and Meta-Analyses (PRISMA) guidelines [10].

The identification of articles for the systematic review was completed with a comprehensive search (Table 1) of titles and abstracts of key electronic databases and additional records. The electronic databases MEDLINE, CINAHL, Embase and SPORTDiscus were systematically searched from inception to March 2020. Broad ranging search terms were agreed on by the authors (JMG, DRB and KBL). The following key scholarly, peer-reviewed journals were also hand searched: *Journal of the American Podiatric Medical Association*, *The Foot*, *Journal of Foot and Ankle Research*, and *Foot & Ankle International*. Grey literature was searched using: grey literature databases, customised Google search engines, targeted websites and consultation with content experts [11], and recent major conference proceedings were searched using the Web of Science Core Collection database through the La Trobe University Library. All titles and abstracts identified from the search were downloaded to Endnote X8 (Thomson Reuters, Philadelphia, Pennsylvania, USA).

**Table 1:** Search strategy

MEDLINE and Embase (Ovid) and CINAHL and SPORTDiscus (EBSCO)	
1.	exp foot orthosis/
2.	foot orthoses.mp
3.	(orthotic* or orthos*s or insole* or heel insert* or ortho* material$ or shoe* or footwear or footwear material$ or sock* or hosier* or shod).mp
4.	1 OR 2 OR 3
5.	(kinetic* or plantar pressure* or peak pressure* or contact area or contact time or maximum force).mp
6.	4 AND 5

### Inclusion and exclusion criteria

Once all duplicates were removed, the titles and abstracts were independently screened by two authors (JMG and GAW). This process determined whether a study was to be included based on the predetermined eligibility criteria (Table 2), while minimising reviewer bias. The list of selected studies was discussed between authors until consensus was achieved. If a consensus was not able to be achieved, a third author (DRB) was engaged to officiate any disagreement regarding inclusion/exclusion status of studies. For the purpose of this review, the eligibility criteria included studies that assessed the effects of flat orthotic materials on plantar pressures compared to a control (shoe alone) condition using in-shoe pressure testing while participants who were free from chronic systemic disease were walking. The eligibility criteria were initially applied to all titles and abstracts, and later to full-text articles if more detail was required. All studies that met the inclusion and exclusion criteria, as well as a previous narrative literature review on the topic [5], had their reference lists hand searched for further included articles. In addition, citation tracking was performed using Google Scholar.

**Table 2:** Eligibility criteria

**Inclusion criteria:**	
• studies that evaluated the effects of flat insoles constructed from different materials on plantar pressures;	
• studies published in English;	
• studies that compared to a control (shoe alone) condition;	
• studies that used ‘in-shoe’ testing apparatus.	
**Exclusion criteria:**	
• studies conducted on animals (non-humans);	
• studies that evaluated cadavers;	
• studies conducted on children (aged under 18 years);	
• studies that evaluated the effects of taping, padding, splinting, bracing, casting, contoured foot orthoses or insoles, or orthopaedic devices defined as other than flat foot orthoses or insoles;	
• studies of activities other than walking;	
• studies where participants had systemic, neurological or inflammatory arthritic pathologies such as diabetes mellitus, cerebrovascular accident, Parkinson’s disease, and rheumatoid arthritis;	
• studies that were not peer-reviewed, scholarly publications of experimental or quasi-experimental research.	

### Methodological quality assessment

All studies accepted for review underwent methodological quality assessment using the Quality Index described by Downs & Black [12]. The Index assesses the quality of non-randomised studies as well as randomised studies [12].

The original Quality Index scale consists of 27 items (maximum score 32) covering four domains: reporting, external validity, internal validity, and power [12]. It has been shown to have high internal consistency (KR-20 = 0.89), test-retest (*r* = 0.88) and inter-rater (*r* = 0.75) reliability, and high criterion validity (*r* ≥ 0.85) [12]. In the original Quality Index, criterion 27, which assesses statistical power, is scored between 0 (insufficient statistical power) to 5 (sufficient statistical power). For the purpose of this review, criterion 27 was modified so that studies assessed as having sufficient statistical power (i.e. provided a power calculation) received a score of 1, and studies that did not provide such detail, or when the statistical power of the study was unable to be determined, received 0. This resulted in the modified Downs and Black Quality Index used in the review maintaining 27 items but having a lower maximum score of 28 compared to the original index. To allow comparability between studies, scores were converted to percentages. Two authors (JMG and DRB), independently scored the included studies. Once all studies were scored, the authors met and discussed any discrepancies until consensus was obtained, at which time a final score was agreed on.

### Statistical analysis

Following methodological assessment, articles were grouped according to materials tested and outcome variables. Data were then synthesised descriptively. Meta-analyses were not conducted because the included studies were not sufficiently homogeneous in terms of participants, interventions, and outcomes. Standardised mean differences (Cohen’s *d*) [13] were calculated in Microsoft Excel using an appropriate formula [14]. Effect sizes were interpreted as negligible (0 to < 0.15), small (0.15 to < 0.40), medium (0.40 to < 0.75), large (0.75 to < 1.10), and very large (> 1.10) [14].

## Results

**Fig. 1:**
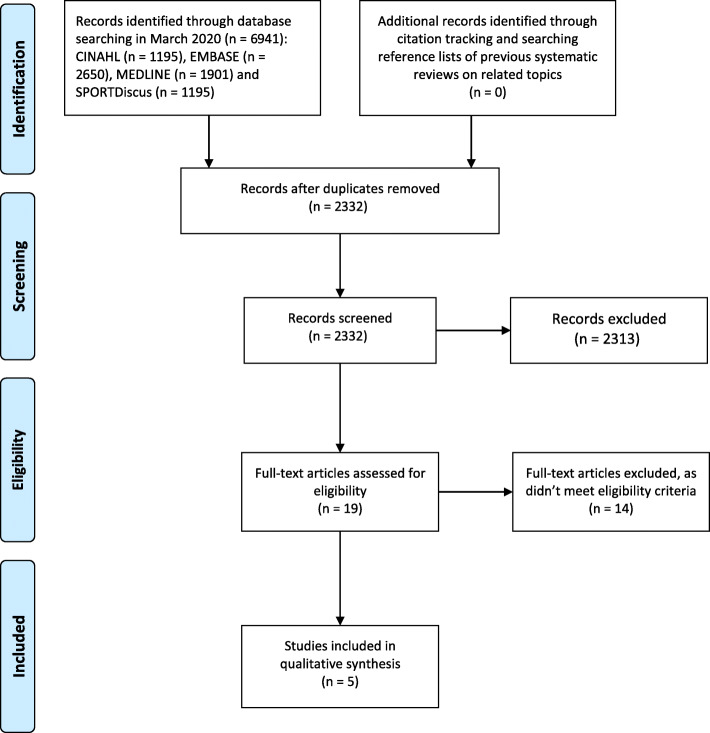
PRISMA flow diagram

The search identified 2332 potential titles and abstracts. Following screening, 19 full-text articles were assessed for eligibility of which 14 were excluded. The remaining five studies were deemed suitable for inclusion (Fig. 1). All the studies used in-shoe analysis to investigate the effect of foot orthotic materials on plantar pressures while walking. Collectively the included studies investigated the following materials: polyurethane (including PORON®), polyethylene (including Plastazote®), ethyl vinyl acetate (EVA) and carbon graphite. All studies were laboratory-based and used a repeated measures design.

### Quality assessment of included studies

The quality of the five included studies only varied slightly, with Quality Index scores ranging from 20 to 23 (Table 3); the full Downs and Black Quality Index with questions is presented in Additional file 1. All included studies [6, 15–18] clearly stated aims, outcome measures, participant characteristics and interventions. The laboratory-based studies were allocated the maximum score for the criterion that addresses confounders due to their repeated-measures study design. However, no laboratory-based studies satisfied the criterion of reporting adverse events, as such detail is rarely documented in studies of this design involving participants that walked distances of only 10 m or less in one session lasting no more than several hours. Two laboratory-based studies [17, 18] tested participants during a single session, so both were assumed to have satisfied the criterion concerning patients lost to follow-up, as there could not be any.

**Table 3:** Modified Downs and Black Quality Index results for each study

Authors, date	Reporting										External validity			Internal validity – bias							Internal validity – confounding						Power	Total (max. 28)	Total %
	01	02	03	04	05	06	07	08	09	10	11	12	13	14	15	16	17	18	19	20	21	22	23	24	25	26	27		
Healy et al., 2012 [6]	1	1	1	1	2	1	1	0	0	1	1	U	1	U	0	1	1	1	1	1	1	1	1	U	1	U	0	20	71
McCormick et al., 2013 [15]	1	1	1	1	2	1	1	0	1	1	1	U	1	1	0	1	1	1	1	1	1	1	1	0	1	1	0	23	82
Rao et al., 2009 [16]	1	1	1	1	2	1	1	0	0	1	1	U	1	U	U	1	1	1	1	1	1	1	1	U	1	U	0	20	71
Rogers et al., 2006 [17]	1	1	1	1	2	1	1	0	1	1	1	U	1	U	0	1	1	1	1	1	1	1	0	0	1	1	0	21	75
Tong & Ng, 2010 [18]	1	1	1	1	2	1	1	0	1	1	1	U	1	U	0	1	1	1	1	1	1	1	0	0	1	1	0	21	75

0 = No, 1 = Yes, U = Unable to be determined (received a score of 0)

For the full criteria of the Modified Downs and Black Quality Index see Additional file 1

All of the five studies did report actual probability values (i.e. *p*-values) and all studies were considered as having used appropriate statistical tests to assess the main outcomes data. As recommended in the Quality Index [12], it was assumed that the distribution of data were appropriate for all statistical tests even when not described, such as in the three studies with relatively small sample sizes (*n* < 11) [6, 17, 18]. Of these studies, only Rogers et al. [17] reported that they checked the data were normally distributed and appropriate for parametric testing.

Regarding internal validity, blinding was reported in one of the studies [15]. This study attempted to blind participants to each material being tested, however none of the included studies blinded assessors. Each laboratory-based study did document the interventions for all measuring sessions. Selection bias was minimised in all included studies by having clearly defined recruitment strategies and eligibility criteria. Three laboratory-based studies randomised the interventions that were tested [6, 15, 16]. All studies used valid and reliable in-shoe plantar pressure measuring systems; the pedar–X® [19–22] or the F–Scan™ [23–26].

Regarding external validity (generalisability), no studies specifically reported whether the participants were representative of populations that these orthotic materials would be used on, however all studies provided a setting that was representative of the environment that patients receiving orthotic therapy would experience.

### The effect of orthotic materials on plantar pressures

Of the five studies included in this review, the following materials were investigated: *polyurethane* (including PORON®) [6, 17, 18], *polyethylene* (including Plastazote®) [17, 18], *ethyl vinyl acetate* (EVA) [6, 15] and *carbon graphite* [16]. In addition to investigating different materials, the studies used a variety of plantar pressure outcome measures and applied a variety of masks to the plantar aspect of the foot, thereby dividing the foot into a range of different anatomical areas. Rao et al. [16] reported major findings only for the medial and lateral midfoot, whereas Rogers et al. [17] reported only the forefoot region, defined as between the base of the hallux and digits and the beginning of the medial arch of the foot. Tong and Ng [18] simply reported both feet as one entity. Healy et al. [6] and McCormick et al. [15] applied similar anatomical masks to the hallux, first metatarsal/1st metatarsophalangeal joint (MTPJ), lateral metatarsals/lateral forefoot, and medial heel and lateral heel. However, Healy et al. [6] combined the midfoot region for analysis, whereas McCormick et al. [15] divided this area into medial and lateral segments.

A summary of the findings from these five studies is presented in Table 4 and Additional file 2.

**Table 4:** Studies using in-shoe testing to investigate the effects flat foot orthoses materials have on plantar pressures during waking

Author, date	Study design/participants/ sample size	Equipment/protocol	Plantar pressure variables of interest	Type of foot orthosis/insole and materials tested	Main findings
Healy et al., 2012 [6]	Laboratory-based study with repeated measures design. ‘Healthy’ participants with mean (SD) age 30.9 (12.4) years, weight 69.3 (12.2) kg and height 172.0 (9.4) cm. *N* = 10 (4 males and 6 females).	F-Scan™ in-shoe system (Tekscan, Boston, USA) Sampling rate 100 Hz. Walking speed: “participants walked on a treadmill at a self-selected speed”, walking speed was then maintained across the subsequent testing conditions. Participants wore ‘standardised plimsoll shoes (a minimalist athletic shoe with a canvas upper and rubber sole).’	Peak pressure (kPa), peak force (N/BW), pressure-time integral (kPa.s) and average contact area (cm^2^).	Conditions: (i) a shoe alone condition (i.e. control), (ii) 3 mm flat insole of low density polyurethane (Shore A hardness 20–25), (iii) 3 mm flat insole of medium density polyurethane (Shore A hardness 55 ± 3), (iv) 3 mm flat insole of low density EVA (Shore A hardness 25), and (v) 3 mm flat insole of medium density EVA (Shore A hardness 50).	Compared to a shoe alone condition, medium density polyurethane insole materials provided significant reductions in peak pressure (kPa) in the first metatarsal region (*p* < 0.05; 215.7 ± 69.8 kPa vs. 180.0 ± 67.2 kPa), as did both the medium and low density polyurethane as well as low density EVA at the lateral metatarsals (*p* < 0.05; 352.5 ± 77.4 kPa vs. 288.0 ± 62.9 kPa, 292.2 ± 51.6 kPa and 295.7 ± 54.8 kPa, respectively). Low and medium density polyurethane materials were most effective at increasing contact area (cm^2^) and reducing pressure time integral (kPa.s).
McCormick et al., 2013 [15]	Laboratory-based study with repeated measures design. Participants with mean (SD) age 25.1 (9.63) years, weight 68.2 (13.8) kg and height 1.70 (0.11) m. *N* = 30 (7 males and 23 females).	Pedar® in-shoe system (Novel GmbH, Munich, Germany) Sampling rate 50 Hz. Walking speed controlled. Participants walked on a walkway in ‘standardised thin cotton socks’ and their most commonly used footwear.’	Peak pressure (kPa), maximum force (%BW) and contact area (cm^2^).	Conditions: (i) a shoe alone condition (i.e. control), (ii) customised polypropylene foot orthosis, (iii) contoured polyethylene sham foot orthosis, (iv) contoured EVA sham foot orthosis, and (v) flat 3 mm EVA sham foot orthosis.	Compared to a shoe alone condition, a flat 3 mm EVA material with a vinyl top cover significantly reduced peak pressures (kPa) at both the medial and lateral heel, mean difference significant at the 0.05 level (Bonferroni adjusted).
Rao et al., 2009 [16]	Laboratory-based study with repeated measures design. Participants with midfoot arthritis, mean (SD), range; age 63 (6), 55–78 years and body mass index 29.7 (5.1), 19.9–38.1 kg/m^2^. *N* = 20 (all participants were female).	Pedar® in-shoe system (Novel Inc., St Paul, MN) Sampling rate 90 Hz. Walking speed controlled. Participants walked over an undescribed surface in ‘subjects’ own footwear.’	Average pressure (kPa), contact time (% stance) and contact area (cm^2^).	Conditions: (i) a shoe alone condition (i.e. control), (ii) shoe with custom moulded ¾ length shoe insert, and (iii) shoe with flat full length insert made of carbon graphite, semi rigid with an average thickness of 1.6 mm.	Compared to a shoe alone condition, a 1.6 mm flat carbon graphite insole provided reductions in average pressure (kPa), contact time (% stance) and contact area (cm^2^) in the medial midfoot and in contact time (% stance) and contact area (cm^2^) at the lateral midfoot.
Rogers et al., 2006 [17]	Laboratory-based study with repeated measures design. Participants with mean age 25 years, mean weight 70.3 kg and mean height 1.73 m. *N* = 9 (2 males and 7 females).	F-Scan™ in-shoe system (Tekscan Inc., Boston, USA) Sampling rate not reported. Control of walking speed: not reported, so likely not controlled. Participants walked on a walkway in undescribed footwear other than it being ‘subjects’ shoes.’	Peak pressure (kPa) and force-time integral (N.s).	Conditions: (i) a shoe alone condition (i.e. control), (ii) flat 6.4 mm thick PORON® insole, and (iii) combination flat 6.4 mm insole consisting of a 3.2 mm Plastazote® top-layer and a 3.2 mm PORON® bottom-layer.	Compared to a shoe alone condition, forefoot peak pressure (kPa) was significantly lower when using a 6.4 mm PORON® insole and a 6.4 mm PORON®/Plastazote® composite insole (*p* < 0.05). No significant differences were found in the force-time integral between the shoe alone condition and the PORON® (*p* = 0.64) and the shoe alone condition and PORON®/Plastazote® combination insoles (*p* = 0.42).
Tong & Ng, 2010 [18]	Laboratory-based study with repeated measures design. ‘Healthy’ participants with mean (2SD*) age 29 (3) years, weight 75.0 (3.7) kg and height 1.75 (0.04) m. *N* = 5 (sex of participants not stated).	F-Scan™ in-shoe system (Tekscan Inc., Boston, USA) Sampling rate not reported. Control of walking speed: not reported other than “…subjects were instructed to walk at their usual walking speed…”, so possibly not controlled. Participants walked on a walkway in undescribed footwear other than it being ‘subjects’ sports shoes.’	Minimum, maximum and peak pressures (kPa).	Conditions: (i) a shoe alone condition (i.e. control), (ii) 6.2 mm Slow Recovery PORON® (extra soft) flat insole, (iii) 6.2 mm PORON® (soft) flat insole, (iv) 6.2 mm PORON® (soft) and firm Plastazote® flat insole, and (v) 6.2 mm PORON® (soft) and soft Plastazote® flat insole.	Compared to a shoe alone condition, a 6.2 mm PORON® and firm Plastazote® combination insole provided significant difference for mean peak contact pressure (kPa) (*p* < 0.03; 60.7 ± 11.3 kPa vs. 47.9 ± 8.4 kPa) which accounted for an approximate 27% mean pressure reduction (whole foot).

Notes: The most relevant information and data from the studies have been provided, *N.s* newton-second, *kPa* kilopascal, *N/BW* newton-body weight, *EVA* ethyl vinyl acetate, *kPa.s* kilopascal-second, *%BW* percentage of body weight, *authors reported 2SD

*Polyurethane* (an open cell foam) was tested by Healy et al. [6] in both 3 mm low and medium densities, with both densities being found to reduce peak pressure and peak force across all regions of the plantar foot compared to a control (i.e. shoe alone) condition. The largest reductions in both outcome measures were under the lesser metatarsal region. Low density polyurethane provided a large reduction in peak pressure and peak force (Cohen’s *d* = 0.97 and Cohen’s *d* = 0.76, respectively) [6]. Similarly, medium density polyurethane provided a large reduction in both peak pressure and peak force (Cohen’s *d* = 0.96 and 0.81, respectively) under the lesser metatarsals [6]. Low density polyurethane also provided a medium reduction in peak pressure (Cohen’s *d* = 0.44) and a small reduction in peak force (Cohen’s *d* = 0.24) at the plantar medial heel [6]. In the same region, medium density polyurethane also provided a medium reduction in both peak pressure and peak force (Cohen’s *d* = 0.44 and 0.42, respectively) [6]. In addition, the effect of polyurethane on pressure-time integral was investigated by Healy et al. [6]. Low density polyurethane also provided a medium reduction in the pressure-time integral at the first metatarsal (Cohen’s *d* = 0.44), a large reduction at the lateral metatarsals (Cohen’s *d* = 0.90), and a medium reduction at the medial heel (Cohen’s *d* = 0.61) [6]. Medium density polyurethane also reduced pressure-time integral across the same plantar foot regions, providing a medium reduction at the first metatarsal (Cohen’s *d* = 0.54), a medium reduction at the lateral metatarsals (Cohen’s *d* = 0.42), and a small reduction at the medial heel (Cohen’s *d* = 0.32) [6]. Low and medium density polyurethane increased contact area across all plantar regions of the foot [6]. Low density polyurethane provided larger reductions under the hallux, first metatarsal, lesser metatarsals and midfoot plantar regions (Cohen’s *d* = 0.79 (large), 0.63 (medium), 0.98 (large) and 0.55 (medium), respectively) [6].

Some types of *polyurethane* are produced and sold under trade names – *PORON®* (Rogers Corporation, Chandler, AZ 85224 USA) is one such commonly used insole material. Tong and Ng [18] found that PORON® and slow release PORON® both provided very large reductions in mean peak pressure across the whole foot compared to a control (i.e. shoe alone) condition (Cohen’s *d* = 1.55 and Cohen’s *d* = 2.01, respectively). Tong and Ng [18] also found that when adding both a *soft and firm Plastazote®* (Zotefoams plc, Croydon, England) to *PORON®*, the combination of materials led to very large reductions in peak pressure across the foot compared to a control (i.e. shoe alone) condition (Cohen’s *d* = 1.52 and Cohen’s *d* = 1.59, respectively). Similarly, Rogers et al. [17] found that PORON® and a combination PORON®/Plastazote® provided very large reductions in peak pressure at the forefoot compared to a control (i.e. shoe alone) condition (Cohen’s *d* = 1.95 and Cohen’s *d* = 1.70, respectively). However, with regards to force-time integral at the forefoot, Rogers et al. [17] found that PORON® and a PORON®/Plastazote® combination only provided negligible and small reductions (Cohen’s *d* = 0.14 and Cohen’s *d* = 0.24, respectively). The thickness of all PORON®, Plastazote® and combination PORON®/Plastazote® insoles tested was 6.2 mm in the Tong & Ng [18] and 6.4 mm in the Rogers et al. [17] study.

Two studies have investigated *EVA* [6, 15] and they both tested 3 mm thickness EVA. Healy et al. [6] tested two densities of EVA, which were reported to be ‘low density’ and ‘medium density’, while McCormick et al. [15] only tested one density, which was 90 kg/m^3^. Regarding peak pressure, Healy et al. [6] found that low density EVA led to a medium reduction (Cohen’s *d* = 0.46) at the first metatarsal and a large reduction (Cohen’s *d* = 0.89) at the lateral metatarsals compared to a control (i.e. shoe alone) condition. Similarly, McCormick et al. [15] found that 90 kg/m^3^ EVA provided a medium reduction of peak pressure (Cohen’s *d* = 0.52) in the medial heel region compared to a control (i.e. shoe alone) condition. Pressure-time integral was also investigated by Healy et al. [6] for low and medium density EVA, but both densities only led to small and negligible effects across all regions of the plantar foot. Regarding contact area, both the low density and the medium density 3 mm EVA tested by Healy et al. [6] and the 90 kg/m^3^ 3 mm EVA tested by McCormick et al. [15] increased contact area. The largest increases in contact area were found with low density EVA beneath the medial heel (Cohen’s *d* = 0.30; small effect) and hallux (Cohen’s *d* = 0.49; medium effect), with medium density EVA beneath the lateral forefoot (Cohen’s *d* = 0.28; small effect) and hallux (Cohen’s *d* = 0.43; medium effect) [6], and with 90 kg/m^3^ EVA beneath the medial midfoot (Cohen’s *d* = 0.87; large effect) and hallux (Cohen’s *d* = 0.61; medium effect) [15].

Finally, *carbon graphite* was tested by Rao et al. [16] in 1.6 mm thickness. In contrast to the other materials that increased contact area under the feet, the use of carbon graphite led to a small reduction in contact area at the forefoot (Cohen’s *d* = 0.33), medium reduction at the midfoot (Cohen’s *d* = 0.47) and small reduction at the heel (Cohen’s *d* = 0.22) compared to a control (i.e. shoe alone) condition [16].

## Discussion

This systematic review has summarised and synthesised the evidence relating to the effect of orthotic materials on plantar pressures while walking shod. All included studies compared orthotic materials in the form of flat insoles to a shoe alone condition by assessing changes in plantar pressures using gold standard in-shoe pressure testing equipment. The methodological quality of the included studies was varied, with McCormick et al. [15] scoring more highly, indicating better internal validity.

Prior to discussing the main findings, several inconsistencies were encountered when reviewing the studies. These inconsistencies are not considered as limitations of the findings of the review, however they do make generalising the findings difficult. These inconsistencies included: variations between studies in thickness of materials tested, most studies omitted standardised durometer hardness values (e.g. Shore values) for the materials tested, and there were differences in masks applied to the plantar surface of the foot for data analysis. Therefore, the conclusions of this review need to be taken into account with reference to the specific thicknesses of material used in each study and that the physical properties of the materials in each study are difficult to compare. In addition, because plantar pressures for different regions of the foot are dependent on mask definition [27], caution is required when comparing data across studies when different masking procedures have been used [28]. A further inconsistency of the included studies was that they did not uniformly report plantar pressure variables. Nevertheless, there is evidence that peak pressure is highly correlated with other plantar pressure variables and some investigators suggest that reporting multiple pressure variables is unnecessary and inefficient [28, 29]. Despite this, and for the sake of broadening our review, we elected to present all plantar pressure variables reported in the included studies, not just peak pressure. Other inconsistencies between studies include the use of different footwear and walking surfaces (e.g. treadmill versus overground), which may limit external validity and should be considered when interpreting findings.

Inconsistencies aside, polyurethane (including PORON®) and polyethylene (including Plastazote®) were found to cause substantial changes in plantar pressures [17, 18]. Polyurethane was found to reduce peak pressure and peak force across all regions of the plantar foot compared to a shoe alone condition [6]. Further, 3 mm polyurethane provided greater reductions in pressure-time integral than 3 mm EVA [6]. Both low and medium density polyurethane increased contact area across all plantar regions of the foot [6]. PORON® is a specific trade name for a version of polyurethane, and it is commonly used in practice. As PORON® is vulnerable to abrasion, it is most commonly used in combination with a protective cover such as vinyl or cambrelle. When compared to a shoe alone condition, PORON® reduced mean peak pressure [18]. Reducing peak pressure is most often achieved by distributing plantar forces over a larger area via increasing plantar contact area [9, 30]. In the case of PORON® this occurs due to its relative softness and its ability to conform to the shape of the foot [9]. As a consequence of its plantar pressure reducing capabilities, PORON® is frequently used as an orthotic material [31]. PORON® and similar materials may, therefore, be considered to reduce plantar pressures in areas where high pressure is contributing to pain. The following examples highlight some of the conditions that this may be considered: in participants with *rheumatoid arthritis*, a significant correlation (*r* = 0.56) has been reported between average pressure and pain beneath the second metatarsal head [32]; in participants with *pes cavus*, a significant correlation has been found between pressure-time integral and foot pain (*r* = 0.49) [33]; and in participants with *degenerative foot disorders*, a significant correlation (*r* = 0.52) has been reported between average pressure beneath the second and third metatarsal heads and pain [34].

PORON® and similar materials may also be indicated for reducing plantar pressures in areas where high pressure can cause tissue break-down, such as with diabetic feet [35]. Raspovic et al. [35] acknowledge that increased plantar pressures often play a significant role in factors leading to tissue breakdown. While it is accepted that sensory neuropathy and ischaemia also contribute to diabetic foot ulceration [36], elevated plantar pressures have been found to contribute the most [37]. A critical threshold pressure value above which ulceration occurs has not yet been identified [38], although mean in-shoe peak pressure at the forefoot of 207 kPa is a suggested target for footwear prescriptions for patients with diabetes with a history of previous foot ulcerations [39]. Nonetheless materials such as PORON® are frequently used in the form of soft orthoses in an attempt to prevent ulcers from forming due to increased plantar pressure [35]. We found evidence in this review to support this practice where studies that evaluated combinations of PORON® and Plastazote® found reductions in peak pressure [17, 18]. Plastazote® readily moulds to the shape of the foot [40], thus distributing the force applied to the plantar foot over a greater area, so this finding is not unexpected. Likewise, PORON®, due to its compressibility, also conforms to the plantar surface of the foot when under load. For this reason, the use of these types of materials in insoles and orthoses [41] is popular among podiatrists, orthotists, and other therapists [18], and is now supported by the evidence we have synthesised in this review.

Like PORON®, the effects of EVA on plantar pressures were found to be substantial, particularly under the medial heel and forefoot [6, 15]. Reduction in peak pressure at the medial heel region is of relevance to plantar heel pain. Reducing pressure under the heel has been suggested to be of benefit for plantar heel pain, particularly when combined with contouring of the orthotic material to produce an orthosis that has a similar shape to the plantar surface of the foot [42]. In addition, reductions at the first and lateral metatarsals hold clinical importance for the treatment of high plantar pressures under the metatarsal heads, for example in the cavus [33] or diabetic foot [35]. Flat EVA (i.e. not moulded) had only a small effect at most on pressure-time integrals across all regions of the plantar foot [6], but this may be clinically important because pressure and time when combined has been postulated to be important in ulcer formation [43–46]. Retrospective studies link high levels of pressure-time integral to plantar ulceration [44], so reducing the combination of pressure and duration that pressure is applied to tissue may be better than reducing peak pressure alone. It is still unknown, however, what reductions in pressure-time integrals are required to reduce the risk of tissue damage/stress related to plantar ulceration [41], but it is plausible that any reduction in pressure-time integral would be advantageous in managing pathology exacerbated by prolonged episodes of sustained pressure. There was no clear consensus for the effect of EVA on maximum force and peak force [6, 15] but EVA, particularly in its lower densities, deforms and contours to the shape of the plantar surface of the foot over time, which increases the contact area that the force is applied to, thus reducing plantar pressure.

The other material included in the review was carbon graphite. Carbon graphite is unlikely to be used as an insole material for plantar pressure reduction, however we included it as it was captured in our search. Carbon graphite had the opposite effect on contact area to the other softer materials; that is, it decreased contact area [16]. This is not surprising as the hardness of carbon graphite in the form of an insole decreases the ability of the inner of the shoe, which is often made from softer and more deforming materials, from being able to conform to the plantar foot and thus, increases contact area. Therefore, an insole made from carbon graphite is not a useful material to use if plantar pressure reduction is the sole aim of the insole.

There are eight limitations of this review that need to be acknowledged. First, the majority of the studies included in the review recruited healthy participants who were free from systemic illness, which limits external validity. Because of this, the results cannot be confidently generalised to clinically-relevant populations such as people with diabetic peripheral neuropathy or rheumatoid arthritis, two populations for whom insoles and foot orthoses are commonly prescribed [47–49]. Second, the majority of included studies had small sample sizes, with three studies [6, 17, 18] having 10 or fewer participants. This may have led to them being underpowered to detect clinically meaningful findings [50, 51]. Third, two of the studies included in the review [17, 18] did not report whether walking speed was controlled for. Walking speed can influence plantar pressure [52], so caution is needed when considering the findings from these two studies. Fourth, comparisons between the pedar–X® and F–Scan™ are difficult as the technology and specifications vary between systems. For example, the systems use different sensor technologies (pedar is a capacitive-based system, whereas F-Scan is a resistive-based system) [53]. In addition, the systems have different sensor resolutions and data collection frequencies. Fifth, although we calculated effect sizes to provide some clinical meaning to the plantar pressure data reported in the included studies (i.e. from a statistical standpoint), a critical threshold or target for plantar pressure reduction that is required to reduce damage (e.g. to prevent ulceration) is still not clear [38]. Sixth, the influence of degradation of the materials due to wear – due to both time and activity – has not been adequately studied, so our review cannot make any conclusions about this issue. Seventh, the review has only considered studies testing orthotic materials in a flat state, as used in a simple insole. Our findings, therefore, do not consider the effect of orthotic materials once they have been altered or manipulated, such as when these materials are heated or added to other materials when manufacturing custom-made contoured foot orthoses. Finally, the review excluded non-English language studies, limiting any contribution from such literature.

When considering all of the limitations listed above, and that the materials included in our review have only been tested in one or two studies, our review provides limited evidence that some orthotic materials reduce plantar pressures. This evidence would be strengthened by further well-designed studies that use gold standard in-shoe pressure testing equipment, test materials commonly used in clinical practice, and recruit participants that are representative of clinically-relevant populations where raised plantar pressures have been found to cause problems (e.g. populations that are older, or those that have diabetes or rheumatoid arthritis). In addition, testing the immediate material effects as well as after periods of wear, and attempting to isolate the effects of orthotic materials in flat and contoured states would be worthwhile.

## Conclusions

Based on the limited evidence supplied from the five studies included in this review, some commonly used orthotic materials can reduce plantar pressures during walking. Polyurethane (including PORON®), polyethylene (including Plastazote®), and EVA were found to provide the greatest reductions in plantar pressures. This evidence can be used by clinicians to guide the materials used for insoles.

## Supplementary information

**Additional file 1.** Full criteria of the Modified Downs and Black Quality Index.

**Additional file 2. **Detailed results of materials tested with Cohen’s *d* effect sizes to provide comparison between studies.

## Data Availability

The datasets used and/or analysed during the current study are available from the corresponding author upon reasonable request.

## Abbreviations

EVA: ethyl vinyl acetate
MTPJ: metatarsophalangeal joint
N.s: newton-second
kPa: kilopascal
N/BW: newton-body weight
kPa.s: kilopascal-second
%BW: percentage of body weight
