# Supplementary material for: Effect of different orthotic materials on plantar pressures: a systematic review
Source: J Foot Ankle Res. 2020 Jun 11;13:35. doi: 10.1186/s13047-020-00401-3 (PMC7291735; doi:10.1186/s13047-020-00401-3)
Supplement: Supplementary file 1 — Additional file 1. Full criteria of the Modified Downs and Black Quality Index. [file 13047_2020_401_MOESM1_ESM.docx]

Additional file 1 Full criteria of the Modified Downs and Black Quality Index

| **Criterion #** | **Description** |
| --- | --- |
| 1 | Is the hypothesis/aim/objective of the study clearly described? |
| 2 | Are the main outcomes to be measured clearly described in the Introduction or Methods section? |
| 3 | Are the characteristics of the patients included in the study clearly described? |
| 4 | Are the interventions of interest clearly described? |
| 5 | Are the distributions of principal confounders in each group of subjects to be compared clearly described? |
| 6 | Are the main findings of the study clearly described? |
| 7 | Does the study provide estimates of the random variability in the data for the main outcomes? |
| 8 | Have all important adverse events that may be a consequence of the intervention been reported? |
| 9 | Have the characteristics of patients lost to follow-up been described? |
| 10 | Have actual probability values been reported (e.g. 0.035 rather than <0.05) for the main outcomes except where the probability value is less than 0.001? |
| 11 | Were the subjects asked to participate in the study representative of the entire population from which they were recruited? |
| 12 | Were those subjects who were prepared to participate representative of the entire population from which they were recruited? |
| 13 | Were the staff, places, and facilities where the patients were treated, representative of the treatment the majority of patients receive? |
| 14 | Was an attempt made to blind study subjects to the intervention they have received? |
| 15 | Was an attempt made to blind those measuring the main outcomes of the intervention? |
| 16 | If any of the results of the study were based on “data dredging”, was this made clear? |
| 17 | In trials and cohort studies, do the analyses adjust for different lengths of follow-up of patients, or in case-control studies, is the time period between the intervention and outcome the same for cases and controls? |
| 18 | Were the statistical tests used to assess the main outcomes appropriate? |
| 19 | Was compliance with the intervention/s reliable? |
| 20 | Were the main outcome measures used accurate (valid and reliable)? |
| 21 | Were the patients in different intervention groups (trials and cohort studies) or were the cases and controls (case-control studies) recruited from the same population? |
| 22 | Were study subjects in different intervention groups (trials and cohort studies) or were the cases and controls (case-control studies) recruited over the same period of time? |
| 23 | Were study subjects randomised to intervention groups? |
| 24 | Was the randomised intervention assignment concealed from both patients and health care staff until recruitment was complete and irrevocable? |
| 25 | Was there adequate adjustment for confounding in the analyses from which the main findings were drawn? |
| 26 | Were losses of patients to follow-up taken into account? |
| 27 | Did the study have sufficient statistical power? |
