# Supplementary material for: Effect of different orthotic materials on plantar pressures: a systematic review
Source: J Foot Ankle Res. 2020 Jun 11;13:35. doi: 10.1186/s13047-020-00401-3 (PMC7291735; doi:10.1186/s13047-020-00401-3)
Supplement: Supplementary file 2 — Additional file 2. Detailed results of materials tested with Cohen’s d effect sizes to provide comparison between studies. [file 13047_2020_401_MOESM2_ESM.docx]

**Additional file 2** Detailed results of materials tested with Cohen’s *d* effect sizes to provide comparison between studies

| **Author,**  **year** | **Materials**  **tested** | **Results (compared to a control/shoe alone condition)** |
| --- | --- | --- |
| Healy et al,  2012 [6] | Polyurethane | Low density polyurethane provided a large reduction in peak pressure and peak force at the lesser metatarsal region (Cohen’s *d* = 0.97 and Cohen’s *d* = 0.76, respectively).  Low density polyurethane provided a medium reduction in peak pressure (Cohen’s *d* = 0.44) and a small reduction in peak force (Cohen’s *d* = 0.24) at the plantar medial heel.  Low density polyurethane provided; a medium reduction in the pressure-time integral at the first metatarsal (Cohen’s *d* = 0.44); a large reduction at the lateral metatarsals (Cohen’s *d* = 0.90); and a medium reduction at the medial heel (Cohen’s *d* = 0.61).  Medium density polyurethane provided a large reduction in peak pressure and peak force at the lesser metatarsal region (Cohen’s *d* = 0.96 and 0.81, respectively).  Medium density polyurethane provided a medium reduction in peak pressure and peak force (Cohen’s *d* = 0.44 and 0.42, respectively) at the plantar medial heel.  Medium density polyurethane reduced pressure-time integral; providing a medium reduction at the first metatarsal (Cohen’s *d* = 0.54), a medium reduction at the lateral metatarsals (Cohen’s *d* = 0.42), and a small reduction at the medial heel (Cohen’s *d* = 0.32).  Low density polyurethane and medium density polyurethane each provided increased contact area across all plantar regions of the foot. Low density polyurethane provided larger reductions at the hallux, first metatarsal, lesser metatarsals and midfoot plantar regions (Cohen’s *d* = 0.79 (large), 0.63 (medium), 0.98 (large) and 0.55 (medium), respectively). |
|  | EVA | Low density EVA led to a medium reduction in peak pressure (Cohen’s *d* = 0.46) at the first metatarsal and a large reduction (Cohen’s *d* = 0.89) at the lateral metatarsals.  Low density EVA increased contact area at the medial heel (Cohen’s *d* = 0.30; small effect) and hallux (Cohen’s *d* = 0.49; medium effect).  Medium density EVA increased contact area at the lateral forefoot (Cohen’s *d* = 0.28; small effect) and hallux (Cohen’s *d* = 0.43; medium effect). |
| McCormick et al, 2013 [17] | EVA | 90 kg/m3 EVA provided a medium reduction of peak pressure (Cohen’s *d* = 0.52) at the medial heel region.  The largest increases in contact area found with 90 kg/m3 EVA were at the medial midfoot (Cohen’s *d* = 0.87; large effect) and hallux (Cohen’s *d* = 0.61; medium effect). |
| Rao et al,  2009 [18] | Carbon graphite | Carbon graphite led to a small reduction in contact area at the forefoot (Cohen’s *d* = 0.33), medium reduction at the midfoot (Cohen’s *d* = 0.47) and small reduction at the heel (Cohen’s *d* = 0.22). |
| Rogers et al,  2006 [15] | PORON® and Plastazote® | PORON® and a combination of PORON®/Plastazote® provided very large reductions in peak pressure at the forefoot (Cohen’s *d* = 1.95 and Cohen’s *d* = 1.70, respectively).  PORON® and a combination of PORON®/Plastazote® provided only negligible and small reductions in force-time integral at the forefoot (Cohen’s *d* = 0.14 and Cohen’s *d* = 0.24, respectively). |
| Tong & Ng,  2010 [16] | PORON® and Plastazote® | PORON® and slow release PORON® each provided very large reductions in mean peak pressure across the whole foot (Cohen’s *d* = 1.55 and Cohen’s *d* = 2.01, respectively).  When adding both a soft and firm Plastazote® to PORON®, the combination of materials led to very large reductions in peak pressure across the foot (Cohen’s *d* = 1.52 and Cohen’s *d* = 1.59, respectively). |
